# Traditional growing rod lengthening without intraoperative neuromonitoring: a 20-year institutional analysis demonstrating neurologic safety and cost savings

**DOI:** 10.1007/s43390-026-01376-0

**Published:** 2026-04-28

**Authors:** Alex R. La Poche, Tyler D. Metcalf, Austin V. Montgomery, Gregory A. Mencio, Jeffrey E. Martus, Craig R. Louer

**Affiliations:** 1 Department of Orthopaedic Surgery, Vanderbilt University Medical Center, Nashville, TN USA; 2 Department of Orthopaedic Surgery, SUNY Downstate College of Medicine, Brooklyn, NY USA

**Keywords:** Early-onset scoliosis, Traditional growing rod, Intraoperative neuromonitoring, Neurologic safety, Cost analysis, Pediatric spine surgery

## Abstract

**Purpose:**

To evaluate the neurologic safety and economic implications of performing traditional growing rod (TGR) lengthening procedures for early-onset scoliosis (EOS) without intraoperative neuromonitoring (IONM). Although IONM is widely adopted for deformity correction, its necessity during routine distraction lengthening procedures remains uncertain.

**Methods:**

A retrospective cohort study was conducted of pediatric EOS patients (< 18 years) who underwent TGR lengthening procedures between 2000 and 2020 at a single tertiary children’s hospital. Inclusion required documented neurologic assessments and radiographic follow-up within 6 months of the index surgery. Patients with alternative fixation strategies or incomplete records were excluded. Demographic, operative, and neurologic outcome data were collected from institutional registries and chart review. The use or omission of IONM was confirmed. Procedural cost estimates for IONM were obtained from institutional billing records using CPT-based rates. Descriptive statistics were calculated in SPSS v29.

**Results:**

Fifty-nine patients (279 TGR lengthening procedures) met inclusion criteria. The mean age at index surgery was 6.6 ± 2.3 years. IONM was not used for any TGR lengthening procedures during this period. No immediate postoperative or delayed neurologic deficits were identified during the 20-year study period. The mean procedure duration was 45.2 ± 6.1 min. The estimated institutional cost of IONM per 45-min TGR lengthening was $6,500, representing an approximate cumulative savings of $1.81 million across all procedures.

**Conclusion:**

Over two decades, no neurologic complications occurred in TGR lengthening procedures performed without IONM, supporting the safety of omitting routine monitoring. Selective IONM use may yield substantial economic benefit without compromising patient outcomes.

**Supplementary Information:**

The online version contains supplementary material available at https://doi.org/10.1007/s43390-026-01376-0.

## Introduction

Distraction-based traditional growing rods (TGR) remain a cornerstone in managing early-onset scoliosis (EOS), offering deformity control while permitting spinal growth through periodic lengthening procedures [1, 2]. Although the risk of neurologic injury during these procedures is presumed to be minimal, any potential complication warrants preventive consideration. Intraoperative neuromonitoring (IONM) is widely employed in pediatric spinal deformity correction to detect evolving neurologic compromise in real time [3]. The Scoliosis Research Society (SRS) recommends IONM for deformity correction surgeries, but whether routine TGR lengthening procedures are considered “deformity correction” is not explicitly stated, and IONM use in these instances remains debatable [4, 5].

Despite its advantages, IONM introduces logistical and financial burdens, including anesthesia modifications, increased operative time, and substantial additional cost, that are compounded by the repetitive nature of lengthening procedures [3]. In addition, there are case reports of electrode burns, compartment syndrome, and necrotizing fasciitis [6–8]. There is also concern about interference of IONM modalities with implanted, programmable devices such as pacemakers, baclofen pumps, and vagal nerve stimulators [9]. Prior studies have shown few, if any, IONM alerts during these procedures, with no lasting neurologic deficits reported [10–12]. However, published data specifically examining outcomes of TGR lengthening procedures performed entirely without IONM are limited, particularly in North America. The present study investigates the neurologic safety and economic impact of performing TGR lengthening procedures without IONM over a 20-year period at a single tertiary pediatric institution.

## Methods

### Study design and population

**Fig. 1:**
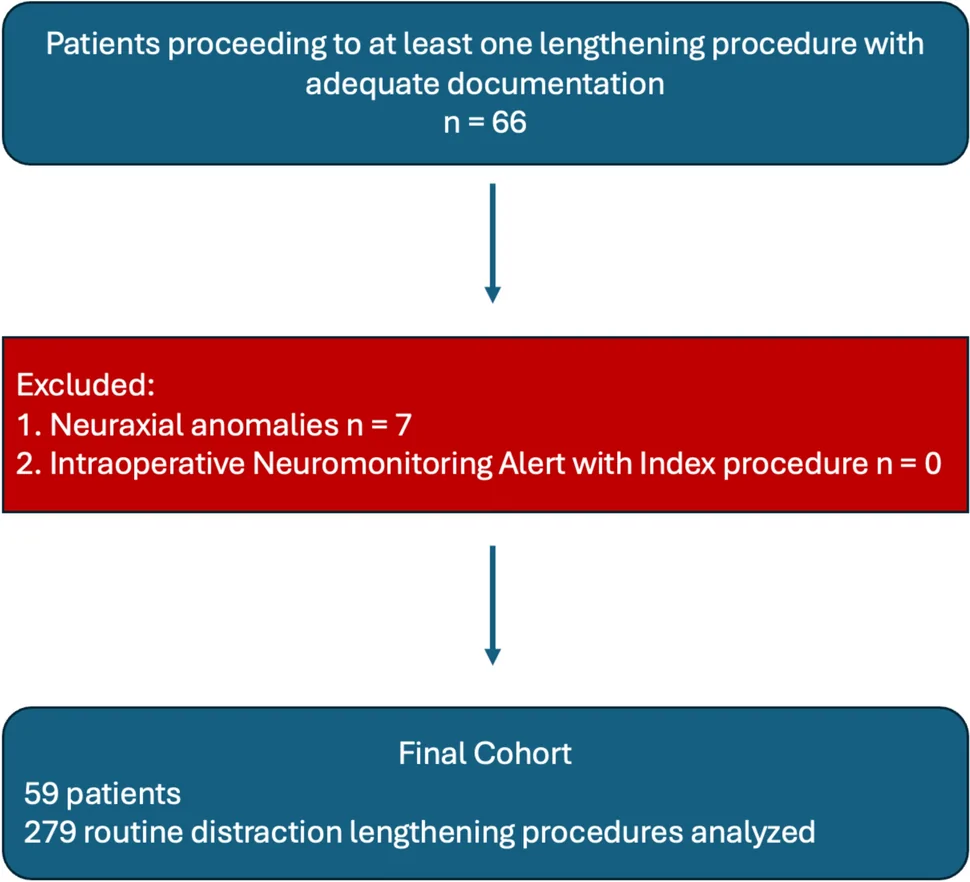
Study cohort selection flow diagram

This IRB-approved retrospective cohort study analyzed pediatric patients (< 18 years) with EOS who underwent TGR lengthening procedures between 2000 and 2020 at a single tertiary children’s hospital. Inclusion required documented neurologic assessments and radiographic follow-up within six months of the index surgery. The patient selection process is illustrated in Fig. 1. Patients with alternative fixation strategies (i.e., guided growth, magnetically controlled growing rods (MCGR)), neuraxial anomalies (tethered cord, syrinx, etc.), or incomplete records were excluded. No neurologic events were observed in this cohort; therefore, formal sensitivity analyses were not performed. Baseline neurologic examination findings and available preoperative imaging were reviewed to establish each patient’s neurologic status prior to lengthening procedures. Preoperative spine MRI was obtained in the majority of patients as part of standard early-onset scoliosis evaluation when clinically indicated. MRI screening was routinely performed for idiopathic, congenital, and syndromic etiologies, while patients with neuromuscular scoliosis (e.g., cerebral palsy) did not routinely undergo MRI because the neurologic etiology was already established. Demographic, operative, and neurologic outcome data were collected from institutional registries and chart review. The use or omission of IONM was confirmed. To minimize heterogeneity in neurologic risk, only routine lengthening procedures were included; procedures involving rod exchange, anchor revision, implant modification, or substantial deformity correction were not classified as routine lengthening procedures and were not included in this cohort, as we regularly utilize IONM for those cases. All cases followed standard TGR lengthening techniques, with spine-based instrumentation using pedicle screw fixation for proximal and distal anchors in the majority of cases; rib-based anchors or alternative fixation constructs were used at the discretion of the treating surgeon.

### Data collection

Patients were identified through an institutional spine registry and verified by chart review. Demographic, operative, and neurologic outcome data were recorded from operative notes and clinical documentation. A delayed neurologic deficit was defined as any new motor weakness, sensory change, or bowel/bladder dysfunction not present immediately postoperatively but documented at a subsequent follow-up visit and attributable to the index lengthening procedure. Routine postoperative follow-up included neurologic examination prior to discharge and at subsequent outpatient visits consistent with institutional early-onset scoliosis protocols, typically occurring within several weeks of each lengthening and at regularly scheduled interval visits thereafter. Neurologic assessments were based on documented standardized clinical examinations performed by the treating surgical team and recorded in the electronic medical record; no additional research-specific neurologic testing was performed. The financial impact of IONM during a TGR lengthening procedure was determined using CPT-based institutional billing rates: G0453 (remote monitoring / 15 min), 95,938 (somatosensory evoked potential study), and 95,939 (transcranial motor evoked potential study). These values reflect institutional billing charges submitted to the payer rather than formal cost-accounting data.

### Statistical analysis

Descriptive statistics were calculated for demographic and clinical variables including etiology, preoperative Cobb angle, comorbidities, operative time, and neurologic outcomes. Cost savings were estimated by multiplying the average per-case IONM charge by the number of lengthening procedures performed.

## Results

A total of 66 patients underwent TGR lengthening procedures with adequate documentation during the study period. Seven were excluded for neuraxial anomalies, leaving a final cohort of 59 pediatric patients who underwent 279 TGR lengthening procedures (Fig. 1). The mean age at index surgery was 6.6 ± 2.3 years. Scoliosis etiology was idiopathic in 32%, syndromic in 25%, neuromuscular in 27%, and congenital in 15% of patients. The mean pre-index major curve was 73.7 ± 21.1°. The average procedure duration was 45.2 ± 6.1 min (Table 1). All patients underwent index TGR placement with IONM, but omission of IONM use in all subsequent routine lengthening procedures was confirmed.

No new motor deficits, sensory changes, or bowel/bladder dysfunction were identified on postoperative examination or during subsequent documented follow-up visits. No delayed neurologic deficits attributable to a lengthening procedure were observed. Because no neurologic injuries were observed, analysis of potential risk factors for such events was not feasible. No lengthening procedures were aborted, modified, or delayed due to intraoperative clinical concerns.

Institutional CPT-based billing data estimated a cost of $6,500 per 45-min IONM session. This estimate reflects institutional billing charges only and does not include professional neuromonitoring fees, additional anesthesia time, operating room opportunity costs, or other indirect expenditures. Based on 279 lengthening procedures, this represents an approximate total cost avoidance of $1.81 million, excluding additional time-related and personnel costs.

**Table 1:** Demographic and clinical characteristics of patients undergoing traditional growing rod lengthening

	Patients undergoing TGR lengthening procedure (*n* = 59)
Age at index surgery, mean ± SD (years)	6.6 ± 2.3
Female, *n* (%)	33 (56.0)
Etiology, *n* (%)	
Idiopathic	19 (32.2)
Syndromic	15 (25.4)
Neuromuscular	16 (27.1)
Congenital	9 (15.3)
Pre-index major curve, mean ± SD (degrees)	73.7 ± 21.1
Comorbidities, *n* (%)	
Developmental delay	21 (37.0)
GI	24 (40.7)
Hyperlaxity	3 (5.1)
Cardiac	15 (25.4)
Musculoskeletal	20 (33.8)
Bladder dysfunction	3 (5.1)
Bowel dysfunction	1 (1.7)
Thoracic insufficiency	1 (1.7)
Upper respiratory infection	6 (10.2)
Asthma	10 (16.9)
Supplemental nutrition	8 (13.6)
Renal	6 (10.2)
Seizures	10 (16.9)
Prior bracing, *n* (%)	30 (50.8)
Procedure time, mean ± SD (min)	45.2 ± 6.1
Neurologic deficit following lengthening, *n* (%)	0 (0)

## Discussion

Neurologic protection during pediatric spine surgery is paramount; however, interventions must balance benefit against cost and procedural complexity. Previous studies using IONM in TGR lengthening procedures report exceedingly low alert and injury rates, suggesting limited incremental value for monitoring in this context [10–18]. Our findings corroborate this literature, demonstrating zero neurologic complications across 279 unmonitored lengthening procedures over two decades.

Sankar et al. reported one IONM alert among 362 lengthening procedures, attributed to a patient with a spinal canal tumor, without subsequent deficit [10]. Similarly, Bess et al. observed a single transient weakness among 633 procedures—this deficit was not associated with an IONM alert and resolved spontaneously [12]. Studies evaluating vertical expandable prosthetic titanium rib (VEPTR) expansions, which impose comparable distractive forces, likewise report no neurologic injuries across thousands of lengthening procedures [13–15]. Collectively, these findings reinforce that the risk of neurologic injury during routine lengthening procedures is exceedingly low. Garg et al. recently reported similar findings in a large Indian cohort of growing rod lengthening procedures performed without IONM, further supporting the safety of this approach [16]. Despite these data, the omission of IONM during routine lengthening procedures has not been widely adopted. The present study represents the first North American series to evaluate this institutional strategy and provides additional evidence supporting the safety of selective IONM omission within a structured protocol and in appropriately selected patients.

Our analysis expands on prior work by demonstrating that omitting IONM in routine TGR lengthening procedures confers substantial cost savings without compromising patient safety. The institutional cost of IONM per short procedure is considerable and indirect costs not calculated here, including OR utilization, anesthesia time, and personnel, further amplify the financial impact. When the baseline neurologic risk approaches zero, the marginal cost-effectiveness of IONM becomes questionable. These findings suggest that selective, rather than routine, IONM use may optimize both safety and value [16–18]. In our practice, selective use denotes reserving neuromonitoring for index implantations, for revision procedures involving re-implantation, anchor modifications, or rod exchange anticipated to produce substantial deformity correction. All routine lengthening procedures in otherwise neurologically uncomplicated patients were performed safely without IONM. However, our findings apply only to patients without known neuraxial abnormalities. Although the cohort included idiopathic, syndromic, neuromuscular, and congenital etiologies, all analyzed patients were at their neurologic baseline at the time of routine distraction lengthening. Therefore, these findings should not be extrapolated to patients with progressive neurologic conditions, known neuraxial abnormalities, or other higher-risk subgroups requiring individualized assessment.

Prior studies have suggested that patients with neuraxial anomalies, such as tethered cord, syringomyelia, or other occult abnormalities, may warrant continued neuromonitoring during growing rod distraction lengthening, and some authors consider these findings relative contraindications to unmonitored procedures [16, 19]. In our practice, documentation regarding IONM decision-making for these patients was inconsistent earlier in the study period; therefore, patients with neuraxial anomalies were excluded from the analysis. Seven patients met these exclusion criteria, four with tethered cords and three with unspecified anomalies. As a result, our findings should not be interpreted to suggest that unmonitored lengthening procedures are safe in this subgroup. Additional study with consistent perioperative monitoring protocols is required before any conclusions can be made about the appropriateness of omitting IONM in patients with neuraxial abnormalities.

Our current protocol parallels certain aspects of the management of magnetically controlled growing rods (MCGR), which are lengthened in the clinic without neuromonitoring; however, operative TGR distraction differs from clinic-based MCGR lengthening in terms of setting, anesthesia exposure, and surgical manipulation. In both settings, neurologic integrity can be assessed immediately following distraction lengthening. Because TGR distraction lengthenings are brief procedures (~ 45 min), a thorough postoperative neurologic examination can reliably identify new deficits, allowing prompt corrective action if needed. This emphasizes that vigilant clinical assessment remains imperative following any spine surgery, including routine lengthening procedures. While our results reinforce the safety of omitting IONM in this context, continued multicenter investigation is warranted to confirm long-term safety and refine criteria for patient selection.

Although rare, IONM is not entirely benign. Reported complications include dermal burns, compartment syndrome from needle electrodes, and severe infections such as necrotizing fasciitis [15–17]. While uncommon, these events underscore that IONM itself carries inherent risk, perhaps further shifting the risk–benefit trade-off in settings where neurologic injury is exceedingly unlikely.

The primary limitations of this study include its retrospective, single-center design, and absence of a contemporaneous monitored comparison group. Additionally, preoperative MRI utilization and other potential modifiers of neurologic risk, including sagittal plane deformity severity and anchor-related complications, were not uniformly captured in this retrospective dataset. As such, although documented neuraxial anomalies were excluded and only routine lengthening procedures were analyzed, granular risk stratification beyond these parameters is limited. Because all patients underwent index TGR implantation with IONM, the analyzed cohort represents a neurologically screened population. Although no patients in this cohort experienced neurologic complications at index implantation, patients with neurologic changes at index surgery would likely not have undergone subsequent unmonitored lengthening procedures. Accordingly, our findings apply specifically to routine lengthening procedures following neurologically uneventful index implantation. The present study did not specifically evaluate the incidence or subsequent management of IONM alerts at index implantation and therefore does not address whether such patients require individualized monitoring strategies during later lengthening procedures. Nevertheless, the study represents one of the largest North American series evaluating TGR lengthening procedures without IONM, with uniform perioperative protocols and consistent neurologic follow-up.

## Conclusion

This 20-year institutional review found no neurologic complications following 279 traditional growing rod lengthening procedures performed without intraoperative neuromonitoring. These results support the safety of omitting routine IONM during planned lengthening procedures in appropriately selected patients without interval neurologic change within our institutional protocol and reaffirm that the neurologic risk associated with standard TGR distraction lengthening procedures is exceedingly low. This study also demonstrates substantial institution-specific healthcare cost savings from selective IONM use. Avoiding routine IONM produced an estimated cost savings of $1.81 million over the study period, exclusive of additional savings from reduced operating time and anesthesia-related expenses. Collectively, these findings suggest that judicious, patient-specific use of IONM can preserve safety while optimizing value and reducing healthcare expenditure. Further multicenter studies are warranted to refine criteria for patient selection and confirm these findings across diverse clinical settings.

## Supplementary Information

Below is the link to the electronic supplementary material.

Supplementary file1 (DOCX 17 KB)

## Data Availability

The data underlying this study are not publicly available due to patient privacy considerations and institutionalrestrictions, as they contain protected health information derived from a single-center retrospective cohort. Datamay be made available from the corresponding author on reasonable request for academic, non-commercialpurposes, contingent upon approval by the Vanderbilt University Medical Center Institutional Review Board andexecution of an appropriate data use agreement.
