# Supplementary material for: Traditional growing rod lengthening without intraoperative neuromonitoring: a 20-year institutional analysis demonstrating neurologic safety and cost savings
Source: Spine Deform. 2026 Apr 28;14(5):1915–20. doi: 10.1007/s43390-026-01376-0 (PMC13553646; doi:10.1007/s43390-026-01376-0)
Supplement: Supplementary file 1 [file 43390_2026_1376_MOESM1_ESM.docx]

**Supplemental Figure 1 –** **Summary of prior studies reporting intraoperative neuromonitoring (IONM) use and neurologic outcomes during growing rod and VEPTR lengthening procedures**

| **PUBLICATION** | **POPULATION** | **Lengthenings** | **IONM Used** | **IONM Alert** | **Neurologic Injury** |
| --- | --- | --- | --- | --- | --- |
| Skaggs, *et al.*  JBJS 2009 | VEPTR - Multicenter | 1185 | 1185 | 1 | 0 |
| Sankar, *et al.*  Spine 2009 | TGR – GSSG Multicenter | 362 | 222 | 1 | 0 |
| Bess, *et al.*  JBJS 2010 | TGR – GSSG Multicenter | 633 | ??? | 0 | 1 – “gait changes”  full recovery |
| Gauthier, *et al.*  Spine Def. 2014 | TGR – CSSG Multicenter | 2193 | 2193 | 0 | 0 |
| LaGreca, *et al.*  JPO 2017 | VEPTR – CSSG Multicenter | 1587 | 1587 | 1 | 0 |
| Garg, B. , *et al.*  Spine Def. 2020 | TGR – Single center | 444 | 116 | 0 | 0 |
| **TOTAL** |  | **5771** | **5303** | **3** | **1** |
| GSSG = Growing Spine Study Group, CSSG = Children’s Spine Study Group. Both later incorporated into the Pediatric Spine Study Group. | | | | | |

1.Skaggs DL, Choi PD, Rice C, et al. Efficacy of intraoperative neurologic monitoring in surgery involving a vertical expandable prosthetic titanium rib for early-onset spinal deformity. J Bone Joint Surg Am. 2009;91(7):1657-1663. doi:10.2106/JBJS.G.00202

2.Bess S, Akbarnia BA, Thompson GH, et al. Complications of growing-rod treatment for early-onset scoliosis: analysis of one hundred and forty patients. J Bone Joint Surg Am. 2010;92(15):2533-2543. doi:10.2106/JBJS.I.01471

3.Sankar WN, Skaggs DL, Emans JB, et al. Neurologic risk in growing rod spine surgery in early onset scoliosis: is neuromonitoring necessary for all cases?. Spine (Phila Pa 1976). 2009;34(18):1952-1955. doi:10.1097/BRS.0b013e3181afe869

4.Gauthier LE, Mandourah Y, Soroceanu A, McIntosh AL, Flynn JM, El-Hawary R. Perioperative Neurologic Injury Associated With Rib-Based Distraction Surgery. Spine Deform. 2014;2(6):481-488. doi:10.1016/j.jspd.2014.08.003

5.LaGreca J, Flynn T, Cahill PJ, et al. Patients Without Intraoperative Neuromonitoring (IONM) Alerts During VEPTR Implantation Did Not Sustain Neurological Injury During Subsequent Routine Expansions: A Retrospective Multicenter Cohort Study. J Pediatr Orthop. 2017;37(8):e619-e624. doi:10.1097/BPO.0000000000000976

6.Garg B, Mohapatra S, Mehta N. Is routine intraoperative neuromonitoring necessary in growing rod lengthening procedures? A retrospective, observational study. Spine Deform. 2020;8(6):1369-1374. doi:10.1007/s43390-020-00163-9
